# Expression and Function of *Scleraxis* in the Developing Auditory System

**DOI:** 10.1371/journal.pone.0075521

**Published:** 2013-09-13

**Authors:** Zoe F. Mann, Weise Chang, Kyu Yup Lee, Kelly A. King, Matthew W. Kelley

**Affiliations:** 1 Laboratory of Cochlear Development, NIDCD, NIH, Bethesda, Maryland, United States of America; 2 Laboratory of Molecular Genetics, National Institute on Deafness and Other Communication Disorders, NIH, Rockville, Maryland, United States of America; 3 Otolaryngology Branch, National Institute on Deafness and Other Communication Disorders, National Institutes of Health, Rockville, Maryland, United States of America; Texas A&M University, United States of America

## Abstract

A study of genes expressed in the developing inner ear identified the bHLH transcription factor *Scleraxis* (*Scx*) in the developing cochlea. Previous work has demonstrated an essential role for Scx in the differentiation and development of tendons, ligaments and cells of chondrogenic lineage. Expression in the cochlea has been shown previously, however the functional role for *Scx* in the cochlea is unknown. Using a *Scx-GFP* reporter mouse line we examined the spatial and temporal patterns of *Scx* expression in the developing cochlea between embryonic day 13.5 and postnatal day 25. Embryonically, *Scx* is expressed broadly throughout the cochlear duct and surrounding mesenchyme and at postnatal ages becomes restricted to the inner hair cells and the interdental cells of the spiral limbus. Deletion of *Scx* results in hearing impairment indicated by elevated auditory brainstem response (ABR) thresholds and diminished distortion product otoacoustic emission (DPOAE) amplitudes, across a range of frequencies. No changes in either gross cochlear morphology or expression of the Scx target genes *Col2A*, *Bmp4* or *Sox9* were observed in *Scx* **^−^** ^*/***−**^ mutants, suggesting that the auditory defects observed in these animals may be a result of unidentified Scx-dependent processes within the cochlea.

## Introduction

The organ of Corti is a neuro-sensory epithelium within the mammalian cochlea that contains the sensory hair cells required for mechanotransduction. Hair cells are precisely patterned during development into a characteristic arrangement of a single row of inner hair cells (IHCs) and three rows of outer hair cells (OHCs). Multiple signaling pathways and developmental cues are required for the correct specification of cell fate and patterning in the inner ear. Our understanding of these pathways has expanded greatly over recent years, yet despite this growing body of knowledge, many additional and as yet unidentified factors are associated with this intricate developmental process [1].

One family of transcription factors well known for their regulatory role in growth and differentiation in a wide variety of cell types and tissues are the basic helix-loop-helix proteins (bHLH). The bHLH proteins are divided into different classes based on their dimerization capabilities, the two most well characterized of which are class A and Class B. Class A bHLH proteins are widely expressed, whereas class B bHLH proteins tend to be tissue-specific [2]. Upon heterodimerization with one another, class A and class B bHLH proteins can bind to consensus E-box sequences in the promoter regions of multiple target genes [2], [3]. A number of class B bHLH proteins including Atonal Homolog 1 (Atoh1) [4], [5], Neurogenic Differentiation 1 (NeuroD1) [6], [7], [8] and Neurogenin 1 [7], [8] have all been shown to play important roles in inner ear development. Here we report on the expression and function of another bHLH, *Scleraxis* (*Scx*) within the inner ear.

Scx is a twist-like bHLH transcription factor that plays an important role in the development of somites and chondrocyte cell lineages [9], [10], force transmitting tendons of the musculo-skeletal system [11], [12], tendons of the middle ear [13] and numerous mesenchymal cell masses [10]. Despite a predominant expression in developing tissues, there are also reports of *Scx* transcripts in adult tissues such as the tongue, diaphragm, limb and cartilage of the bronchi [10]. These findings led to the hypothesis that Scx expression in mature tissues could signify the presence of connective tissue or connective tissue-like cells [10].

Scx is known to regulate *Bmp4* transcription by binding directly within the *Bmp4* promoter region [14]. In addition, Scx also acts cooperatively with Sox9 to enhance the transcription of Sox9-mediated target genes such as *Collagen, type 2 alpha 1*(*Col2a1)* [15]. These Scx targets are intriguing, as all three are expressed in the developing cochlea. *Bmp4* is expressed in cells located directly adjacent to the developing sensory domain, which later become the Hensen’s and Claudius cells of the outer sulcus region [16], [17]. In addition, disruption of *Bmp4* alters the production of hair cells during cochlear development [16], [18], [19]. Sox9 is dynamically expressed in the mesenchymal cells of the developing inner ear where it regulates the differentiation and expansion of otic fibrocytes, the formation of the otic capsule and coiling of the cochlear duct [20]. Col2A1 is also widely expressed throughout the cochlea in mammals [21], [22], [23] and birds [24]. Col2A expression has been documented in both ectodermally and mesodermally derived inner ear structures including the otic capsule, the spiral ligament, the spiral limbus and modiolar connective tissue [21] systems and comprises a major component of the basilar membrane [25], [26] and tectorial membrane [27], [28]. Therefore, we examined the expression of *Scx* and functional consequences of *Scx* deletion on cochlear development and function.

## Experimental Procedures

### Mutant mice and genotyping

*Scx* **^−^** ^*/***−**^ mice were kindly provided by Ronen Schweitzer (Oregon Health and Science University, Portland Oregon). Mice were maintained as heterozygotes on a C57BL/6 mixed background and were bred to generate Scx**^−^** ^/**−**^, Scx**^−^** ^/+^ and *Scx^+/+^* (WT) littermate controls. ABRs were measured at P0 and P25. Mice were genotyped as described by Murchison *et al*., 2007. All animal care and experimental protocols were approved by the joint National Institute of Deafness and other Communication Disorders (NIDCD) and National Institute of Neurological Disorders and Stroke (NINDS) Animal Care and Use Committee.

### ABR and DPOAE measurements

ABRs were performed at postnatal day 25, by placing three sub-dermal needle electrodes; one at the forehead and one at each mastoid location. ABR thresholds were averaged using an alternating polarity click stimulus, as well as 8, 16, and 32 kHz tone bursts. Threshold search began with administration of a 110 dB SPL signal for the click, 8, and 16 kHz stimuli, and a 100 dB SPL signal for the 32 kHz tone burst. The stimulus intensity was decreased subsequently in 10 dB steps, followed by 5 dB steps at lower intensities near threshold, to determine the exact threshold of the response. Thresholds were considered the lowest intensity at which a replicable neurogenic response was identified. DPOAEs were recorded with an ER-10C (Etymotic Research) speaker-probe assembly using the DP2000 DPOAE measurement system, version 3.0 (Starkey Laboratories). ABR stimuli were generated with the auditory-evoked Intelligent Hearing Systems (IHS) software and produced through a high-frequency, ear-specific closed field transducer. DPOAE and ABR data were collected on both ears of each animal. DPOAEs were recorded by placing the speaker-probe assembly in the external auditory canal of the animal. Two primary tones at a frequency ratio (f2/f1) of 1.2 were presented at L1  =  65 dB SPL and L2  =  55 dB SPL. Calibration of the primary tones took place *in situ.* The primary tones were varied in one-eleventh octave steps from 5297 to 10641 Hz, based on the frequency limitations of the speaker probe assembly.

### Immunohistochemistry

Cochleae were dissected from WT, *Scx* **^−^** ^/+^ and *Scx* **^−^** ^/**−**^ littermates and processed either as whole mounts or cryosectioned at a thickness of 12 µm. For whole mount staining, cochleae were fixed in the bullae in 4% paraformaldehyde (PFA) overnight at 4°C, and then washed in 0.1 M phosphate buffered saline (PBS). For cryosectioning, tissue was fixed overnight in 4% PFA at 4°C and subsequently washed with sucrose of increasing concentration (5%, 10%, 15%, 20% and 30%). Tissue was incubated overnight in 30% sucrose at 4°C before embedding in OCT compound (Tissue-Tek). *Scx* expression patterns were visualized using cochlear sections or whole mounts from the Scx-GFP reporter mouse. Cochlear tissue was further stained with primary antibodies against the hair cell-specific protein Myosin VI (Myo6) (Proteus BioSciences, Inc.) (1∶1000, overnight at 4°C), the prosensory marker Sox2 (Millipore Bioscience Research Reagents, 1∶1000 overnight 4°C) or Alexa Fluor-546 phalloidin (Invitrogen, 1∶200, 1h at room temperature) to visualize filamentous actin. To clearly visualize Scx-GFP, tissue was counterstained with the anti-GFP antibody (1∶1000, overnight at 4°C). To visualize primary antibody localization, tissue was subsequently stained with the appropriate Alexa Fluor conjugated secondary antibody (Invitrogen, 1∶1000, 1h at room temperature). Images were acquired with a Zeiss 510 LSM confocal microscope using a 20x Plan Apochromat (NA 0.8) or 40x Plan-Neofluar (NA 1.3) objective. Alexa Fluor-488 secondary conjugates and phalloidin were excited at 488 nm and 546 nm respectively and emitted fluorescence captured using BP 505–530 nm and LP 560 nm emission filters.

### In situ hybridization

Inner ear tissue was dissected and fixed in 4% PFA overnight at 4°C. Tissue was subsequently washed for 30 minutes in 0.1 M PBS before cryoprotection with sucrose (10% – 30% solutions diluted in 0.1 M PBS + 0.02% tween 20) and OCT. Frozen tissue sections were cut at a thickness of 12 µm. Complimentary digoxigenin-labeled RNA probes were generated using published mouse RNA sequences for *Sox9* and *Col2a* (Open Biosystems). The *Bmp4* probe was kindly provided by Doris Wu (NIDCD, NIH). *In situ* hybridization was performed as described previously by [17].

### RNA isolation and first strand cDNA synthesis

Wild type, *Scx* **^−^** ^/+^ and *Scx* **^−^** ^/**−**^ cochleae were dissected and total RNA was extracted using the RNAqueous®-Microkit (Ambion). First strand cDNA synthesis was performed using the SuperScript®III first strand synthesis kit (Invitrogen) using random priming with 500 ng of template.

### Real time quantitative PCR

Real-time PCR was performed using an ABI Prism 7000 real-time PCR machine (Applied Biosystems). All reactions were performed using the qPCR™ core kit for SybrGreen® (Applied Biosystems). Real-time PCR on test samples was carried out in triplicate using appropriate forward and reverse primers for *Sox9*, *Bmp4* and *Col2a*. The sequences for primers (Invitrogen) were as follows: *Sox9* forward 3′-CTGAAGGGCTACGACTGGAC-5’, reverse 5′-GTACTGGTCTGCCAGCTTCC-3′; *Bmp4* forward 3′-CCCAGTGAGGAAAACAAGGA-5′, reverse 5′-TGTTTGCAGCATCCAGGTAG-3′ and *Col2a* 3′-forward CTCGGGGCGAGCGAGGTTTC-5′ reverse 5′-CAGGAGTGCCAGGGAGGCCT-3′. Calculated amounts of cDNA represent expression levels of *Sox9*, *Bmp4* and *Col2a* in cDNA prepared from 500 ng of total RNA. Inter-sample variation was corrected for by normalizing gene expression levels to internal levels of the house-keeping gene large ribosomal protein (RPLPO).

## Results and Discussion

### Scx expression in the developing inner ear

**Figure 1:**
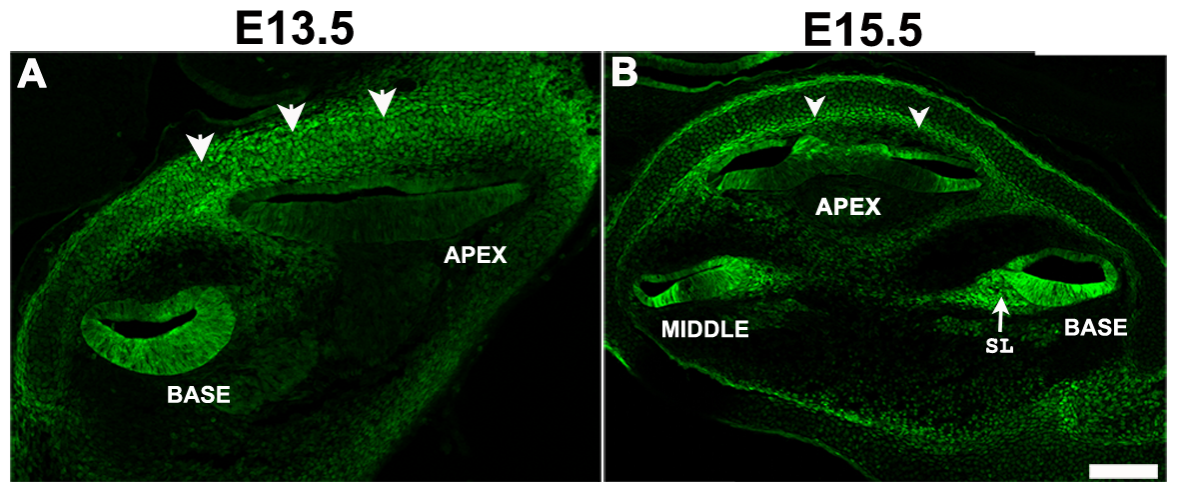
*Scx* is expressed in both mesenchymal and epithelial cells within the inner ear. Images show maximal confocal z-projections through cross-sections of the inner ear from *Scx-GFP* mice at E13.5 (A) and E15.5 (B). At E13.5 *Sc*x is expressed in the developing otic capsule, in otic fibrocytes (white arrow heads) and throughout the basal turn of the cochlear duct. Expression of *Scx* in the middle and apical cochlear turns is noticeably lower. By E15.5 *Scx* expression is notable in the developing spiral limbus (SL) of the basal turn as well as in all four turns of the cochlear duct. While broad expression persists in the basal turn, expression in the remaining turns is more restricted with the most intense expression localized to the lateral floor and ceiling of the duct. *Scx* expression is also still evident in otic fibrocytes (arrowheads). Scale bar is 100 µm.

**Figure 2:**
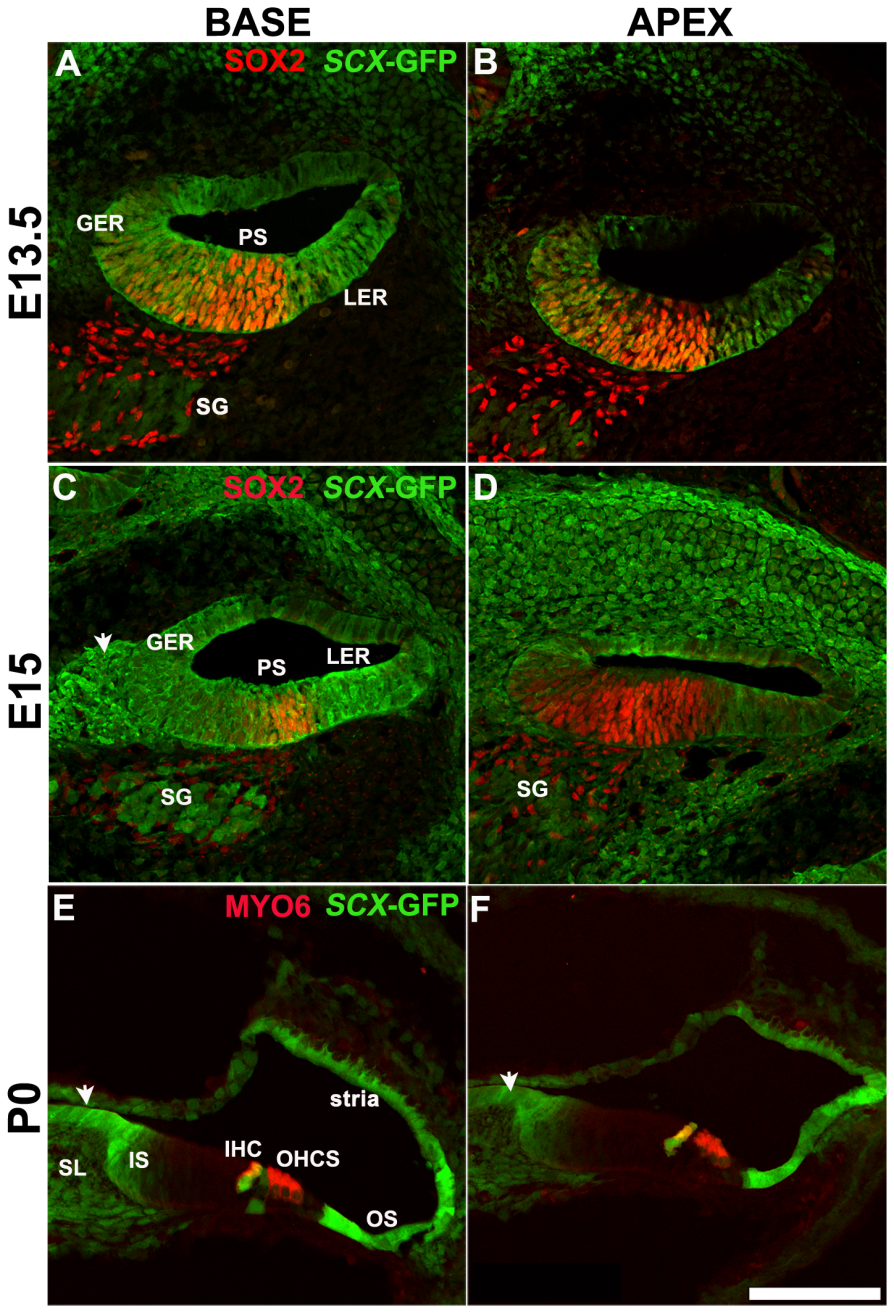
*Scx* expression in the developing mouse cochlear duct. (**A,B**) Images show maximal confocal z-projections through E13.5 cochlear sections showing broad expression of *Scx*-GFP throughout the cochlear epithelium (green). Note the gradient in expression levels, in particular in the roof of the duct, between base and apex. The prosensory region (PS), flanked by the developing greater and lesser epithelial ridges (GER and LER), is marked based on expression of SOX2 (red). Auditory glia within the spiral ganglion (SG) is also present for Sox2. (**C, D**) Maximal z-projections taken through the cochlear duct at E15. *Scx*-GFP expression is present in fibrocytes and the developing otic capsule, most of the cochlear duct, spiral ganglion neurons, and the future spiral limbus. Expression still appears to be more intense in the base, consistent with a base-to-apex gradient of expression (**E,F**) Maximal z-projections through the cochlear duct at P0. *Scx*-GFP expression is restricted to the outer sulcus (OS), developing interdental cells (white arrows), medial region of the inner sulcus (IS), spiral limbus (SL), stria vascularis and the inner hair cells (IHCs). Occasional pillar cells also express *Scx* and weak expression was observed in Reissner’s membrane. The pattern of Scx expression is similar in both the basal and apical regions, indicating that the base-to-apex gradient in *Scx* expression is no longer present. Scale bar is 100 µm

**Figure 3:**
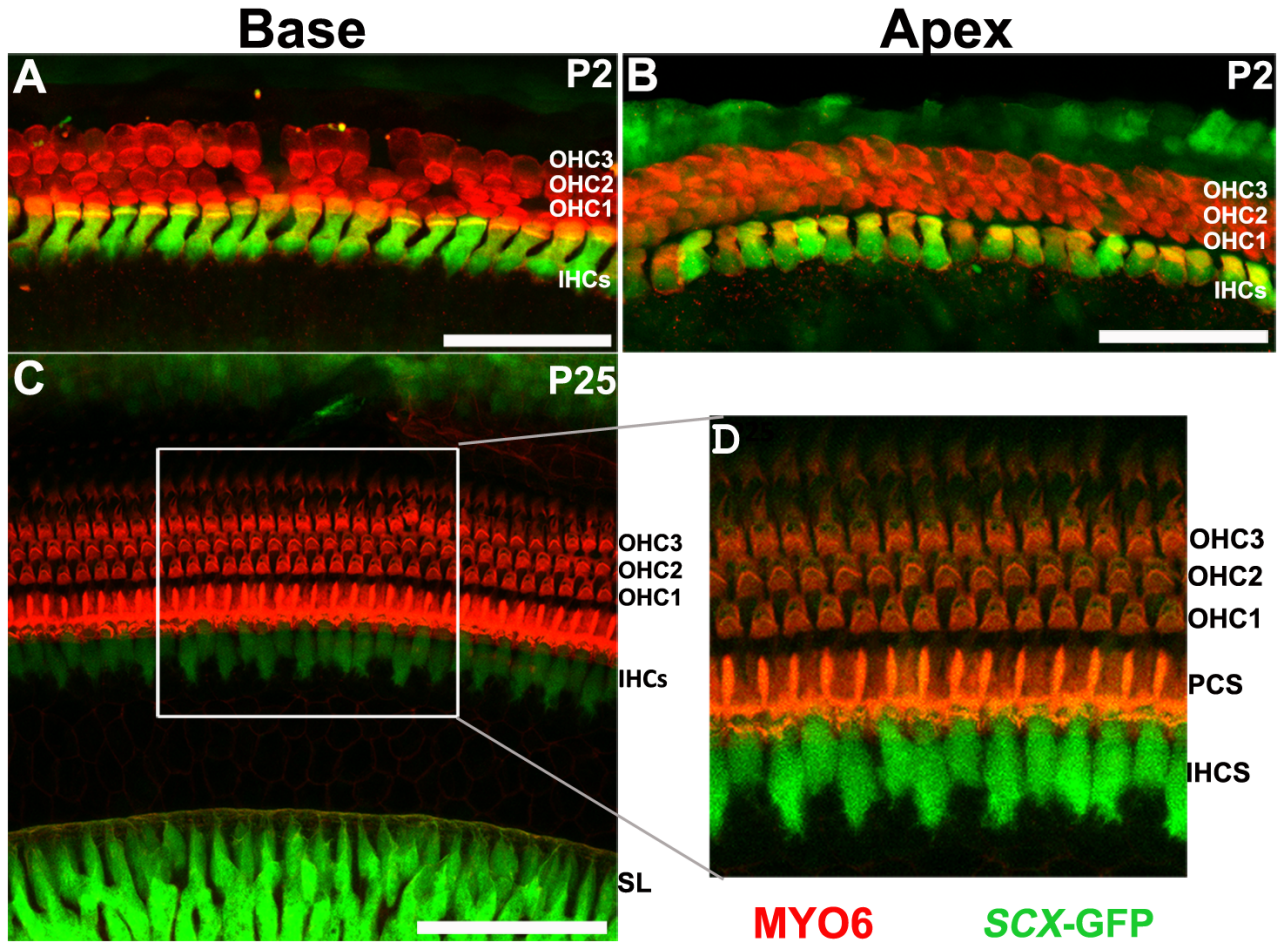
*Scx*-GFP expression the neonatal and adult cochlea. (**A,B**) Maximal confocal z-projections through basal and apical cochlear whole-mounts at P2. *Scx*-GFP expression is shown in green and Myosin 6 in red. By P2, *Scx*-GFP is restricted primarily to the inner hair cells IHCs. However in the apical turn expression of *Scx* is also present in the Hensen’s cells adjacent to the third row of outer hair cells. (**C**) Confocal z-projection through a cochlear whole mount at postnatal day 25 (P25). *Scx*-GFP expression is restricted to the inner hair cells (IHCs) within the cochlear epithelium and is most prominent in the interdental cells of the spiral limbus (SL). Little expression is observed in the outer hair cells (OHCs). Scx expression is also still observed albeit at lower levels in the Claudius/Hensen’s cell region. Inset (**D**) highlights the restriction of *Scx*-GFP to the IHCs. Scale bars are 50 µm.

To determine which cell types within the cochlea express *Scx,* and at which developmental time points, we used a transgenic *Scx-GFP* reporter mouse, which was previously shown to accurately indicate the activity of the *Scx* promoter [29]. This mouse model was generated by cloning of the GFP gene into the genomic region of *Scx* exon 1, in which the majority of coding sequence resides. Resultantly, GFP expression is driven by *Scx* regulatory sequences in a pattern that closely ressembles that of endogenous *Scx* expression [29]. In addition, the ability of *Scx-GFP* to accurately convey activity of the *Scx* promoter has been confirmed by *in situ* hybridization [13]. Figure 1 shows cross sections through the cochlear duct at embryonic day 13.5 (E13.5), a time point prior to hair cell and supporting cell differentiation, and at E15.5 when cells in the cochlea are post mitotic and cellular differentiation has begun [30], [31], [32]. At both time points *Scx* expression was observed throughout the epithelium of the cochlear duct and in the otic mesenchyme (Figure 1A, B). Within the cochlear duct, *Scx* was expressed in a base-to-apex gradient in that a more intense and widespread expression pattern was observed in the basal cochlear turn at both E13.5 and E15.5. Since differentiation of hair cells and supporting cells also occurs in a basal-to-apical gradient [33], [34], we compared *Scx* expression patterns with those of prosensory cell markers or those of differentiated hair cells. Expression of *Scx* was compared with the prosensory marker, Sox2 (red in Figure 2A-D) at E13.5 and E15.5, and with the hair cell differentiation marker Myosin VI (Myo6) at P0 (red, Figure 2E-F). Though overlapping within the prosensory domain, *Scx-GFP* was expressed in a considerably broader area than Sox2, in both the base and apex of the E13.5 cochlea (Figure 2A-B). At the base, *Scx* expression appeared to encompass the entire duct while in the less mature apex, expression was absent in the lateral roof of the cochlear duct in cells that will give rise to the stria vascularis. In addition, weaker expression of *Scx* was also observed in a subset of cells within the developing spiral ganglion. Whilst the specific identity of these cells was not determined, the fact that they did not express Sox2 suggests that they are neurons rather than auditory glia. The base-to-apex gradient in Scx-GFP expression was found to become less pronounced with developmental age (Figure 2A-D), which could result from a more significant downregulation in *Scx* expression at the cochlear base compared to the apex (Figure 2C-F). By E15.5 the expression pattern, but not intensity of *Scx* at the cochlear apex appeared comparable to that observed in the base at E13.5. Down-regulation of *Scx* continued along the length of the duct throughout embryonic development such that by P0, expression of *Scx* within the organ of Corti was restricted to the IHCs and occasionally the inner pillar cells. Strong expression also persisted in the Claudius/Hensen’s cells of the outer sulcus region, the stria vascularis and the interdental cells of the spiral limbus (Figure 2 E-F, Figure 3A-B). In addition, expression of *Scx* was present in the mesenchymal cells of the developing spiral limbus. Finally, the pattern of *Scx* expression was similar at both the base and apex at P0 indicating a loss in the base-to-apex gradient (Figure 2). In contrast with embryonic time periods, the *Scx* expression pattern within the cochlea was consistent between P0-P25 (Figures 2, 3) suggesting that the spatial pattern of *Scx* expression is mature by early postnatal stages. In whole mounts of the cochlea at P2 and P25, the highest *Scx* expression levels were observed in the IHCs of the organ of Corti (Figure 3) and the interdental cells of the spiral limbus (Figure 3C). Expression of *Scx* in interdental cells and the spiral limbus is particularly intriguing as these cells are rich in extracellular matrix components including *Col2A1* and cartilage-specific proteoglycans [35], [36], suggesting that *Scx* could play a role in mediating development of the cartilage/tendon like aspects of these cells. The specific role of Scx in inner hair cells is less clear as there is no evidence that these cells are more cartilage/tendon like than their outer hair cell neighbors.

### Scx ^−/−^ mice display elevated ABR thresholds and diminished DPOAEs

**Figure 4:**
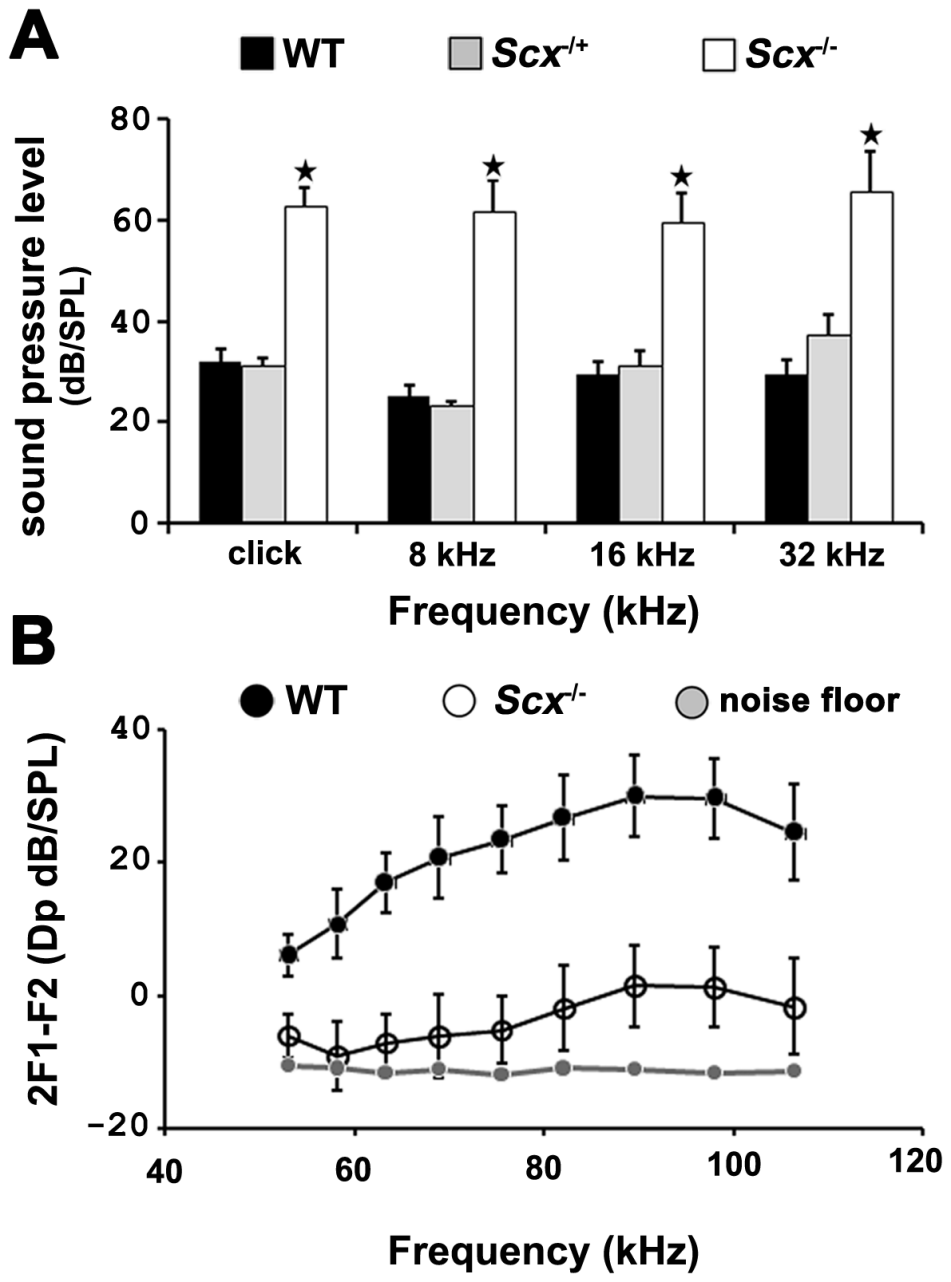
Loss of *Scx* causes elevations in ABR threshold responses and diminished OAEs. (**A**) Auditory brainstem response thresholds in wild type (black), heterozygous (grey) and *Scx*-null (white) mice. ABR thresholds were significantly elevated in *Scx*-null animals (n  =  10) across all frequencies tested when compared to WT mice (n = 5). No significant hearing loss was observed in heterozygous animals (n =  9). Data are mean ± sem. P < 0.05, Student’s *t-*test. (**B**) Mean distortion product otoacoustic emission (DPOAE) amplitudes measured across a range of frequencies in wild type (black) and *Scx*-null (white) mice. DPOAE levels were significantly reduced in *Scx*-null (n = 10) animals compared to WT (n = 5) littermates. Data are mean ± SEM.

Given the expression of *Scx* in auditory hair cells, we hypothesized that its expression would be required for auditory function. To determine if this is the case, ABR thresholds and DPOAEs were analysed at post natal day 25 in *Scx^+/+^*, *Scx* **^−^** ^*/+*^ and *Scx* **^−^** ^/**−**^ mice. We observed a significant (p <0.05 Student’s *t*-test) elevation in ABR threshold across all frequencies tested (40 dB/SPL, 31 dB/SPL, 31 dB/SPL and 8, 16 and 32 kHz respectively) in *Scx* **^−^** ^/**−**^ mice (Figure 4A). However, in accord with the *Scx* phenotype reported by Wang et al (2011), we observed an incomplete penetrance of the hearing loss. Normal hearing (an ABR threshold of between 20-25 dB SPL) was evident in 30% of *Scx* **^−^** ^/**−**^ mice (n = 10). In contrast, no differences in ABR thresholds were observed in any WT or *Scx* **^−^** ^/+^ littermates. To determine whether the hearing phenotype originated at the level of the auditory nerve or within the cochlear sensory epithelium we also assessed DPOAEs in WT, *Scx* **^−^** ^*/+*^ and *Scx* **^−^** ^/**−**^ mice. The DPOAE amplitude was significantly reduced across all frequencies tested in *Scx* **^−^** ^/**−**^ mice (Figure 4B), although with similar incomplete penetrance, but was unaltered in *Scx* **^−^** ^*/+*^ animals (data not shown). DPOAEs reflect integrity of the cochlea itself, in particular the cochlear amplifier (OHCs) and the motor components of cochlear function [37]. These results, along with expression of Scx throughout the developing cochlear duct, suggest a role for Scx in the normal development of one or more aspects of the inner ear. However, we cannot rule out a significant contribution of conductive hearing loss related to defects arising in the middle ear of Scx mutants [13]. Future studies using tissue specific Cre driver mouse lines would be required to differentiate between the roles of *Scx* in middle versus inner ear development and function.

### Gross cochlear morphology appears normal in Scx^−/−^ mice

**Figure 5:**
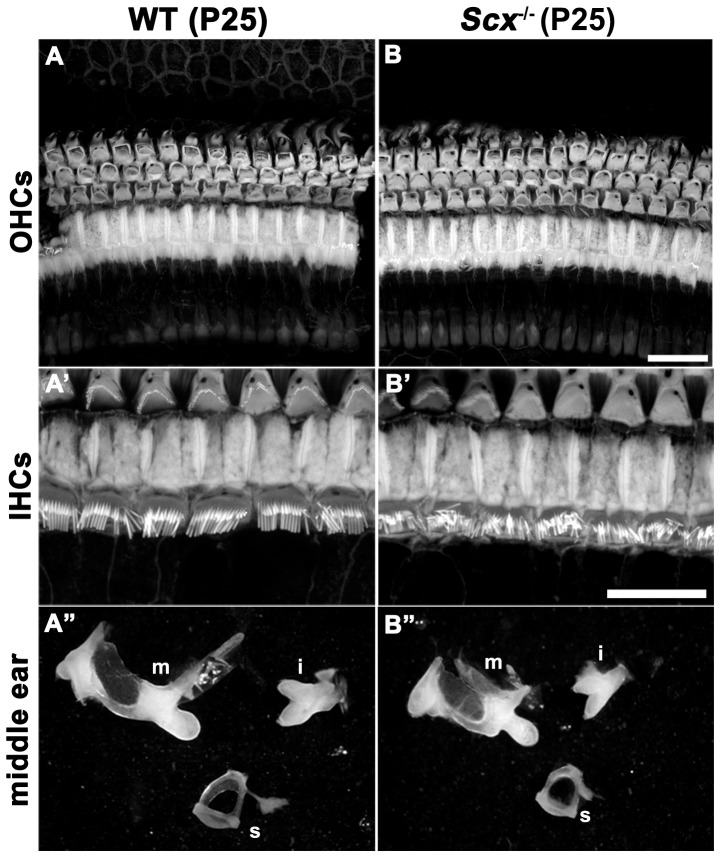
Gross morphology of the inner ear appears normal in *Scx* ^−/−^ mice. (**A-B**) Phalloidin staining in P25 whole-mounts depict the gross cochlear morphology in wild type and *Scx* **^−^** ^/**−**^ mice. (**A**) Maximum z-projection showing the gross morphology of the cochlear sensory epithelium at the level of the outer hair cells (OHCs) in a wild type litter mate. (**B**) Z-projection showing gross cochlear morphology at the OHC level in an *Scx* **^−^** ^/**−**^ mice. (**A’**) **Maximal** z**-**projection showing phalloidin staining at the level of the inner hair cells (IHCs) in the wild type cochlear epithelium. (**B’**) Phalloidin staining at the IHC level in Scx**^−^** ^/**−**^ mice. (**A’’-B’’**) Show the morphology of the middle ear bones in wild type and *Scx* **^−^** ^/**−**^ respectively. Malleus (m), incus (i), stapes (s). Scale bars are 20 µm.

Given the hearing impairment observed in *Scx* **^−^** ^/**−**^ animals and the ability of *Scx* to potentially modulate multiple genes required for cochlear development, we hypothesized that the loss of *Scx* would lead to morphological changes at the cellular level within the cochlea. We therefore compared the cochlear morphologies in WT and *Scx* **^−^** ^/**−**^ littermates at P25. As indicated by phalloidin staining, no obvious differences in gross cochlear morphology were evident at the level of the OHCs (Figure 5A-B) or IHCs (Figure 5A’-B’). Given the central role *Scx* plays in the development and differentiation of chondrocyte cell lineages, we also analysed middle ear bones from wild type and *Scx* **^−^** ^/**−**^ mice. In concurrence with the phenotypes observed in the cochlea, analysis of the malleus, incus and stapes from wild type and *Scx* **^−^** ^/**−**^ litter mates revealed no obvious difference in middle ear bones, apart from the slight reduction in size in *Scx* **^−^** ^/**−**^ animals. We attributed the difference in size of the middle ear bones to the fact that *Scx* **^−^** ^/**−**^ mice are noticeably smaller than wild type litter mates, a finding also reported by Wang et al., 2011.

### Scx-E47: a potential role in differentiation and function of otic fibrocytes

**Figure 6:**
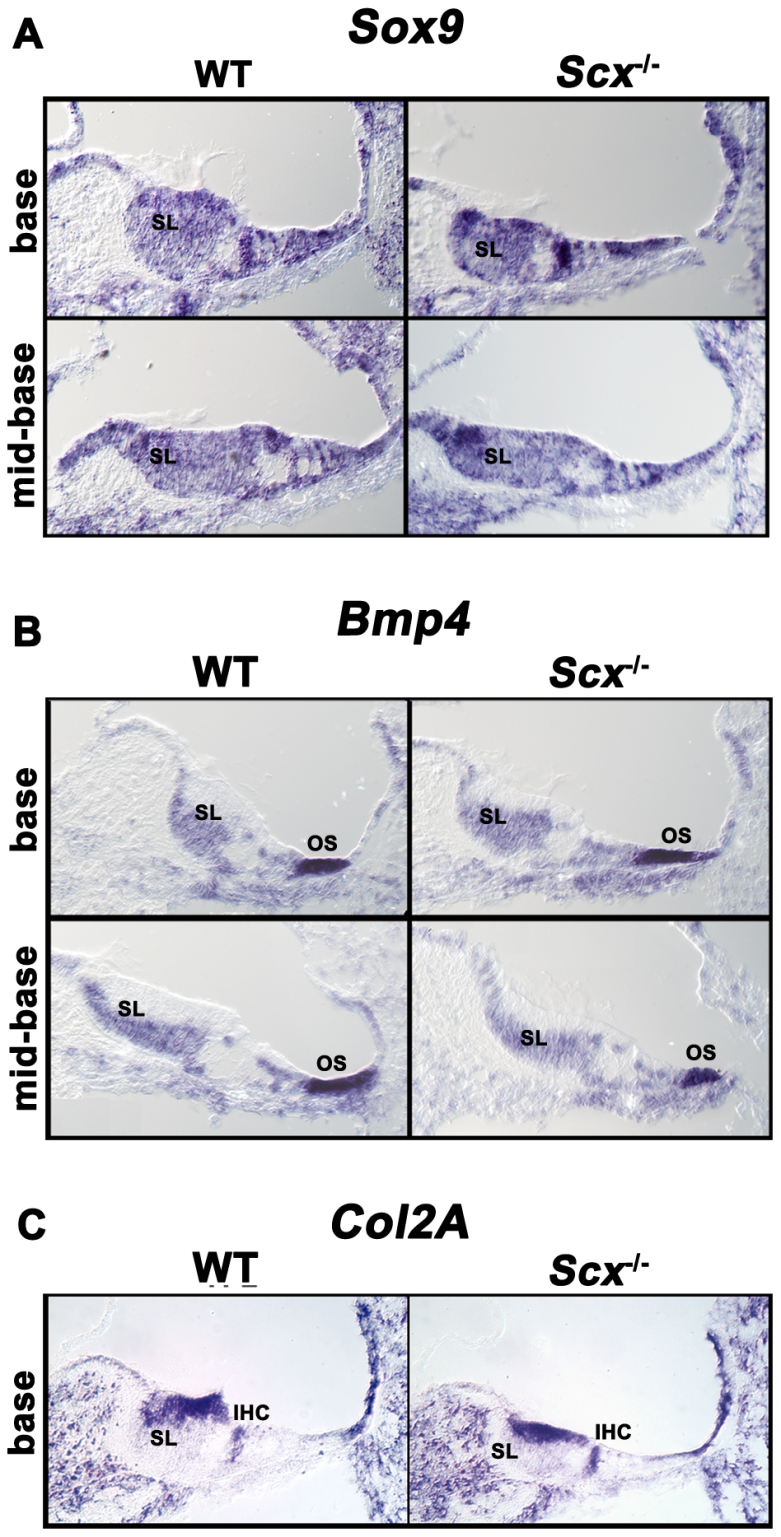
Expression patterns of *Sox9*, *Bmp4* and *Col2A1* appear unaltered in Scx^−/−^ mice. *In situ* hybridization analysis in P2 cochlear cross sections taken from wild type and *Scx* **^−^** ^*/***−**^ littermates. Images show representative expression patterns for (**A**) *Sox9*, (**B**) *Bmp4* and (**C**) *Col2a*. The spiral limbus (SL), outer sulcus (OS) and inner hair cells (IHC) are indicated.

**Figure 7:**
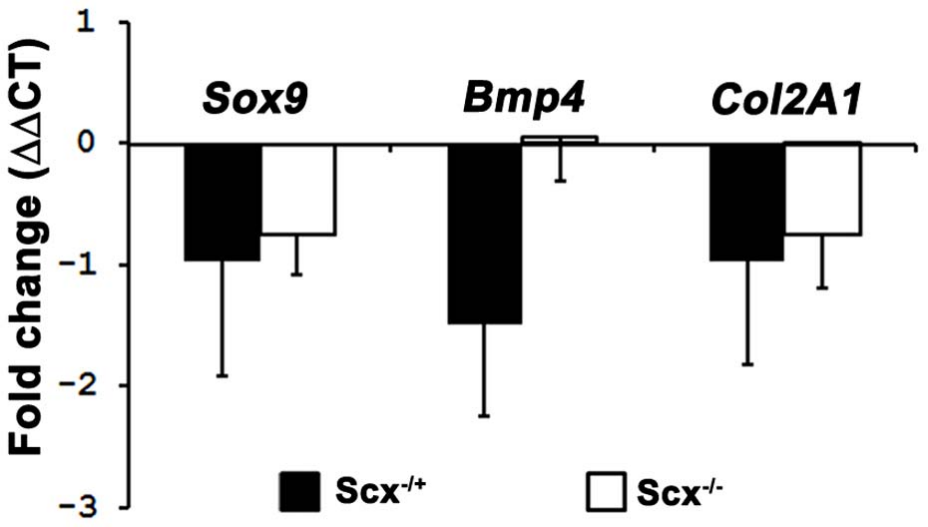
Expression levels of *Sox9, Bmp4 and Col2a* are altered in *Scx* ^−/−^ mice. Graph shows qRT-PCR analysis of *Sox9, Bmp4 and Col2a* mRNA expression levels in heterozygous (*Scx* **^−^** ^*/+*^) (black bars) and Scx null (*Scx* **^−^** ^*/***−**^) P3 (white bars) littermates. Expression levels have been normalized to those in wild type (*Scx^+/+^*) animals Data are mean ± SEM, Student’s *t*-test. P  =  > 0.05 for *Col2A1, Sox9*

Similar to other bHLH proteins, *Scx* carries out its biological functions as a heterodimer with the E2A gene products [10], [15], [38], [39]. The Scx-E47 heterodimer is known to regulate transcription levels of *Collagen 1A1 and 2A1(Col1A1 and Col2A1)* [38], [40], *Bone Morphogenetic Protein 4 (BMP4)* [14] and *Sry-type HMG box 9 (Sox9)* [15], all of which are expressed in the developing cochlea [16], [17], [20], [35], [41], [42], [43]. Given the normal morphology of the organ of Corti in *Scx* **^−^** ^/**−**^ mice and the expression pattern of *Scx* throughout the cochlear duct, we considered that loss of *Scx* could lead changes in the formation and differentiation of otic fibrocytes through its ability to cooperatively modulate *Sox9*-mediated transcription [15]. Sox9 is expressed throughout the periotic mesenchyme during cochlear development where it is required for the expansion and differentiation of otic fibrocytes, formation of the endochondal capsule and epithelial morphogenesis [20]. Furthermore, disruption of Sox9-mediated pathways has previously been linked with defects in the spiral limbus and overlying interdental cells [20], both cell types in which *Scx* is highly expressed (Fig. 3). Therefore, disruption of the network regulating *Sox9*-mediated transcription i.e. the Scx-E47-p300 complex [15] could alter the differentiation state of otic fibrocytes resulting in their impaired function and consequently a dysregulation of inner ear fluid composition and volume. To test this hypothesis, we used in situ hybridization and real-time quantitative PCR (qPCR) to analyse the expression levels of *Sox9* in the cochlea (Figures 6 and 7). In situ hybridization for *Sox9* in P2 cochlear sections indicated no obvious differences in *Sox9* expression between WT and *Scx* **^−^** ^/**−**^ mice (Figure 6A). However, qPCR analysis indicated a consistent, though not significant, decrease in *Sox9* expression in both *Scx* **^−^** ^/+^ and *Scx* **^−^** ^/**−**^ mice compared to controls (Figure 7). Overall, a negative (ΔΔCT) fold change of at least –0.5 was observed in *Sox9* expression, in three out of four *Scx* **^−^** ^/**−**^ mice analysed for qPCR. However, similar to ABR and DPOAE measurements, large variations in the total expression level of *Sox9* were observed between animals. We also investigated *Scx*-induced changes in *Bmp4*, as *Scx* is known to directly regulate *Bmp4* expression levels in chondrocytes during bone ridge patterning [14]. In the developing cochlea, *Bmp4* is important for development of cochlear supporting cells [16], specification of hair cell number [18], [19] and for correct establishment of the vestibular system [17], [43]. However, as was the case for *Sox9*, variability between samples resulted in no significant changes in *Bmp4* expression between *Scx* **^−^** ^/**−**^ and WT animals (Figure 6B, Figure 7). There were also no obvious vestibular phenotypes in any of the *Scx* **^−^** ^/**−**^ animals as indicated by the absence of circling or head bobbing (data not shown). As discussed, previous reports have demonstrated defects in middle ear tendon formation and possibly development of middle ear bones [13]. Similar to the variability in hearing loss, a range of phenotypes was also observed in middle ear tendons of *Scx* **^−^** ^/**−**^ mice [13]. However, the specific expression of *Scx* in cells within the inner ear suggests a role for Scx in these cells as well.

It is important to consider the incomplete penetrance of the hearing phenotype as well as the variability in the expression levels of *Sox9* and *Bmp4*. While it is not possible to correlate unaffected hearing loss with normal levels of *Sox9* and *Bmp4* in the same animals, the comparable rates of occurrence for each of these events suggests that the two parameters may be related. The basis for this variability is unclear, however the *Scx* **^−^** ^*/***−**^ mouse model used in this study was maintained on a mixed genetic background. Therefore, the incomplete penetrance of both the hearing and molecular phenotypes might be explained by the presence of a genetic modifier segregating independently of *Scx*. Genetic modifiers are known to give rise to variable phenotypes by modifying the expression of other genes, and can manifest at the molecular or cellular level (Nadeau, 2001). In our experiments, it is possible that a strain-specific genetic modifier is regulating the activity or expression of a protein downstream of the Scx transcription factor, and ultimately, lessening the hearing and molecular phenotypes observed in *Scx* **^−^** ^/**−**^ animals. The generation of multiple *Scx* null mouse strains with differing isogenic backgrounds would be necessary to explore this hypothesis further. Finally, the reduced ABR thresholds observed in *Scx* **^−^** ^/**−**^ mice have recently been linked with the occurrence of otitis media [13]. Whilst one cannot disregard the involvement of otitis media in Scx associated hearing loss, we think it unlikely given the absence of any obvious defects in the cochlear sensory epithelium a common sequela of acute and chronic otitis media [44], [45], [46].

### Scx could contribute to the development and maintenance of collagen-rich structures within the inner ear

The Scx-E47 heterodimer is known to regulate expression levels of *Col1A1* and *Col2A1* [38], [40]. The collagen protein family encompasses a large number of extracellular matrix molecules, which assemble to form supramolecular structures with great functional diversity [47]. Col2A is a fibril forming collagen that provides tensile strength in a variety of tissues [48] including cartilage [47], the eye [49], [50] and the cochlea [23], [27], [28]. Col2A is present in abundance throughout the cartilaginous matrix sitting beneath the sensory epithelium [51] and is a major component of the cochlear tectorial membrane (TM) [23], [27], [52], a structure essential for the process of hearing [53]. The TM is a ribbon-like extracellular matrix structure, which sits directly above the mechanosensory hair cells [51]. The OHC bundles are embedded within the TM and are stimulated in response to the shearing that occurs between the TM and the reticular laminar during sound stimulation [51]. The mammalian TM contains two distinct fibril systems. One consisting of collagen fibrils that run radially across the TM and a second comprised of a tectorin-based matrix arranged as a striated sheet. This precise arrangement of TM fibrils is thought necessary for correct auditory function [54]. Since, the TM is anchored at the spiral limbus, a major site of *Scx* expression, and within close proximity to the interdental cells, which are thought to secrete components contributing to the structure of the TM [55], auditory defects in *Scx* **^−^** ^/**−**^ mice could result from defects in *Col2A1* expression. To determine whether Scx plays a role in the regulation of *Col2A1* expression in the cochlea, we analysed *Col2A1* expression using in situ hybridization and quantitative real-time PCR. In situ hybridization revealed no significant differences in *Col2A1* expression between WT and *Scx* **^−^** ^*/***−**^ animals. Analysis of *Col2A1* expression by means of qPCR revealed an overall reduction in expression of *Col2A1* in *Scx* **^−^** ^*/+*^ and *Scx* **^−^** ^*/***−**^ animals, however again due to the high levels of variation between samples this difference was not significant (Figures 6 & 7). Previous work has shown that an optimum balance between *Scx* and *Sox9* regulates *Sox9*-dependent *Col2A1* expression. Therefore, if levels of *Scx* fall sufficiently beneath those of *Sox9* both *Sox9*-mediated *Col2A1* transcription and thus *Col2A* expression levels are perturbed. The precise mechanisms regulating this transcriptional network are unclear, however if in *Scx* **^−^** ^/+^ animals *Scx* levels drop sufficiently below *Sox9*, this may be sufficient to alter Sox9-mediated *Col2a* transcription and thus account for the down-regulation of *Col2A1* in both *Scx* **^−^** ^/+^ and *Scx* **^−^** ^/**−**^ cochleae.

Throughout development, bHLH transcription factors play key roles in the differentiation and patterning of multiple tissues. The inner ear is no exception relying on a delicate balance between multiple bHLH transcription factors, such as Atoh1, Ngn1, NeuroD and Hes5, to establish correct cellular differentiation and patterning. Scx strongly influences expression of extracellular matrix components including collagens, tenascin and fibronectin [10], [11], [12], [56]. In addition, twist bHLH transcription factors are known to regulate epithelial-to-mesenchymal transitions (EMT) in multicellular tissues during development [57]. In *Drosophila*, twist promotes EMT by up-regulating mesenchymal markers including fibronectin, vimentin and smooth muscle actin, whilst simultaneously downregualting epithelial markers such as E-cadherin, α and γ catenin [57]. The early expression of *Scx* in the otic mesenchyme could also indicate a role for the transcription factor in fibrocyte differentiation and gross cochlear morphogenesis. Mesenchymal cells originate from the paraxial mesoderm and begin to condense around the otocyst at E10. Mesenchymal cells closest to the developing otic epithelium differentiate to become otic fibroblasts, whilst those further away condense, aggregate and differentiate to from the cartilaginous cells of the otic capsule. Successful inner ear development relies on a series of complex interactions between the ectodermal mesenchyme and epithelia of the cochlear duct [33], [58], [59]. Mesenchymal-epithelial interactions throughout cochlear development are also required for otic fibrocyte differentiation and gross cochlear morphogenesis [20]. To date, there are limited data available regarding the precise molecular signalling pathways that coordinate these events. The early expression of *Scx* in the otic mesenchyme is consistent with a role for the transcription factor in fibrocyte differentiation and gross cochlear morphogenesis. Mesenchymal cells originate from the paraxial mesoderm and begin to condense around the otocyst at E10. Mesenchymal cells closest to the developing otic epithelium will differentiate to become otic fibroblasts, whilst those further away will condense, aggregate and differentiate to from the cartilaginous cells of the otic capsule. Successful inner ear development relies on a series of complex interactions between the ectodermal mesenchyme and epithelia of the cochlear duct [33], [58], [59]. Mesenchymal-epithelial interactions throughout cochlear development are also required for otic fibrocyte differentiation and gross cochlear morphogenesis [20]. To date, there are limited data available regarding the precise molecular signalling pathways that coordinate mesenchymal-epithelial interactions in the developing tissues of the inner ear.

An additional factor involved in mesenchymal-epithelial transitions is transforming growth factor beta (TGF-β) [60]. TGF-β2 has been implicated in such tissue interactions during cochlear development, most noticeably in the cells of the spiral limbus and overlying interdental cells [61], where we demonstrate robust *Scx* expression. In tendons, TGF-β is also involved in the conversion of mechanical force into biochemical signals through up-regulation of *Scx* [62]. As *Scx* mRNA levels and *Scx*-E-box binding activity are modulated by TGF-β [63], [64] it is possible that *Scx* regulates mesenchymal-epithelial interactions in the cochlea via a TGF-β mediated pathway. EMT is important for proper differentiation of otic fibrocytes, which in turn is required for gross morphologicalchanges.Regulation of cochlear EMT by TGF-β signalling would imply a role for this pathway in the differentiation of otic fibroblasts and in regulating gross changes in cochlear morphology throughout development. In the TC6 chondrocyte cell line, *Scx* expression is regulated by both bone morphogenetic proteins (BMPs) and TGF-β [64]. At early postnatal stages, *Scx* expression is present in cells of the outer sulcus region in which expression of BMP4 has recently been shown [16] and in the spiral limbus, where TGF-β [61] is thought to play an important developmental role. As the expression profiles of both BMPs and TGF-β in the adult cochlea are not known, it is possible that at later stages, *Scx* expression is maintained by an additional factor.

Another underlying cause for the hearing loss observed in *Scx* **^−^** ^/**−**^ mice could be defects in the otic fibrocytes of the spiral ligament, spiral limbus and/or stria vascularis, all of which exhibit robust *Scx* expression between P0-P25 (Figures 2, 3). Fibrocytes within the spiral limbus are interconnected via gap junctions, which are thought to be essential for potassium cycling during mechanosensory transduction [65]. In addition, fibrocytes also express numerous ion [66], [67] and aquaporin [68] channels and extracellular matrix proteins [69], [70], [71]. Moreover, alterations in fibrocyte integrity, caused by changes in expression of fibrocyte-specific genes are associated with numerous inner ear pathologies. These include Pendred syndrome [72] and age-related hearing loss [73], [74]. Similar to the phenotype observed in *Scx* **^−^** ^/**−**^ mice, defects in cochlear fibrocytes result in a moderate hearing loss (30-40 dB) with no obvious morphological defects at the level of the cochlea [75]. In this model of hearing loss, dysregulation of the ionic composition and normal fluid volume within the middle ear is correlated with impairment of efficient sound transmission [75]. A similar comparison could be drawn in models of cystic fibrosis, where mutations in the cystic fibrosis transmembrane conductance regulator (CFTR) lead to defects in fluid secretion and absorption [76]. One could therefore hypothesize that the hearing deficits observed in *Scx* **^−^** ^/**−**^ mice at P25 may become more pronounced in aged mice. Unfortunately, because of defects in tendon formation *Scx* **^−^** ^/**−**^ mice exhibit severe movement defects that become increasingly pronounced with age. As a consequence, it is difficult to maintain these animals until late adult stages. Again, generation of a conditional knockout specific to the inner ear would be useful to determine a possible role for Scx in age-related hearing loss.

In summary, we report the expression pattern of and functional role for the bHLH transcription factor *Scx* in the developing inner ear. Initially, *Scx* is broadly expressed in both mesenchymal and epithelial cells of the inner ear but progressively becomes restricted to specific cell types, in particular cells within the spiral limbus and organ of Corti. In addition, functional assessment of auditory function indicates a significant hearing loss in *Scx* mutants. However, examination of possible morphological and molecular targets of Scx signaling within the cochlea did not indicate any specific changes. Previous results have suggested that hearing loss in *Scx* mutants may be a result of changes in the middle ear, but the robust expression of *Scx* in several cell types within the cochlear duct suggest additional roles for Scx. For instance, in the developing skeletal system and in tendons and ligaments, Scx is associated with the formation of a correct extracellular matrix necessary for function of these cell types [10], [11]. Our data support the idea that cells of the spiral limbus closely resemble those of tendons, ligaments and cells from the chondrogenic lineage [35] in that they possess a connective tissue matrix that can provide tension to the BM. Furthermore, the numerous ways in which Scx contributes to *Col2A* expression supports the hypothesis that the interdental cells of the spiral limbus are involved in secretion and maintenance of TM components. No obvious differences in the thickness or gross morphology of the BM were observed in *Scx* mutants, but it is possible that loss of *Scx* alters the precise alignment of single collagen fibrils within the BM matrix, leading to subtle changes in cochlear mechanics. A more detailed analysis of BM mechanics and ultrastructure would be required to address this hypothesis. Finally, several of the molecular analyses conducted in this study were limited because of the high degree of variability in *Scx* mutants. These results suggest that uncharacterized genomic modifiers may regulate the inner ear effects of Scx signaling, a hypothesis that can only be tested by moving the *Scx* mutant allele onto a pure genetic background.
